# Editorial: Inherited retinal dystrophies: a light at the end of the tunnel?

**DOI:** 10.3389/fcell.2023.1301279

**Published:** 2023-10-04

**Authors:** Brian D. Perkins, Glenn P. Lobo, Altaf A. Kondkar, Jose M. Millan

**Affiliations:** ^1^ Department of Ophthalmic Research, Cole Eye Institute, Cleveland Clinic, Cleveland, OH, United States; ^2^ Department of Ophthalmology and Visual Neurosciences, University of Minnesota Medical School, Minneapolis, MN, United States; ^3^ Department of Ophthalmology, College of Medicine, King Saud University, Riyadh, Saudi Arabia; ^4^ Molecular, Cellular and Genomics Biomedicine, La Fe Health Research Institute, Valencia, Spain

**Keywords:** retinal dystrophy, inherited retinal degeneration (IRD), gene therapy, retinal regeneration, genetic testing, photoreceptor degeneration

Inherited retinal dystrophies (IRDs) are a Research Topic of more than two dozen diseases affecting rod and cone photoreceptors and the retinal pigment epithelium (RPE) of the retina. Patients with IRDs suffer from progressive vision loss that may lead to blindness resulting from retinal degeneration. Building off the revolutionary advances in human genetics knowledge over the past 30 years, hundreds of mutations in more than 280 unique disease genes responsible for IRDs have been identified. The clinical and genetic complexity of IRDs complicates efforts to make rapid and accurate clinical diagnoses and to develop effective therapeutic treatments for IRDs. The 11 selected papers in this special Research Topic entitled “*Inherited Retinal Dystrophies: A light at the end of the tunnel?*” highlight the development of novel stem cell and animal models to study disease progression, advances in genetic screening technologies, and biochemical studies that reveal disease mechanisms that are all central to finding treatments and the eventual cures for patients suffering from IRDs. Included are review articles by Chew and Iannaccone discuss advances in stem cell therapies, optogenetics, and retinal prosthetics as therapeutic options and by Miller et al.that explain how epigenetic changes contribute to photoreceptor death in IRDs.

A major challenge to clinicians is to accurately diagnose patients with IRDs based on clinical presentations and to combine this information with diagnostic results from genetic testing services. While whole-genome sequencing (WGS) and whole-exome sequencing (WES) can identify genetic variants, the cost and necessary infrastructure makes these technologies prohibitive for wide-spread use. Diagnostic genetic testing by WGS or WES in the United States can cost several thousand dollars per patient. To address this problem, Panneman et al. describe a panel of single molecule Molecular Inversion Probes (smMIPs) that cost $30 per sample. This smMIPs panel targets exons and splice sites for all known genes associated with Retinitis Pigmentosa (RP) and Leber congenital amaurosis (LCA). The group discussed the outcomes of using this smMIPs panel to screen almost 1,200 probands and compared the effectiveness of this approach with that of WES.

Determining whether novel genetic variants identified by WGS and WES are causal for pathogenicity also remains a significant obstacle for accurate disease diagnosis. In particular, genetic variants that occur in elements that regulate RNA splicing often require functional assays to validate and confirm the effect of these variants. Rodriquez-Hidalgo et al. describe the identification of two candidate variants in the *ABCA4* gene and subsequent functional assays to determine pathogenicity. Similarly, Fernandez-Suarez et al. used WGS to identify a novel genetic variant in the gene encoding thyroid hormone receptor beta (*THRB*) as a disease-associated variant for a family with a dominantly-inherited form of cone dystrophy. Subsequent genetic analysis revealed this pathogenic variant was also found in individuals with Stargardt disease and macular dystrophy, thus expanding the possible clinical outcomes of genetic variants in *THRB*. These papers highlight the utility of WGS to identify novel variants, but also the need for functional testing to confirm pathogenicity.

Once genetic variants are deemed pathogenic, additional work is necessary to explain disease mechanisms. Jones et al. describe the complementary use of human pluripotent stem cell retinal organoids and preclinical mouse models to investigate the role of *DRAM2* in cone-rod dystrophy. The *DRAM2* gene encodes a transmembrane protein with poorly defined cellular function. Mutations in *DRAM2* lead to a rare form of age-related maculopathy but the details of disease progression remained unclear. The authors discuss how different disease models can be leveraged to study disease progression and the advantages and caveats of making comparisons between human stem cell-derived organoids and mouse models.

Three papers describe efforts to uncover the molecular mechanisms driving photoreceptor dysfunction and vision loss. Sadeh et al. explored the impact of 10 distinct disease-causing missense mutations in the *CACNA1F* gene, which encodes the Ca_v_1.4α_1_ calcium channel. Using a combination of molecular modeling, patch-clamp analysis, and protein stability assays, the authors discovered that mutations in this calcium channel altered protein structure and decreased ion current through the mutant channels. Importantly, they discovered that the mutant proteins were degraded by the proteosome and that inhibition of proteosome machinery could partially restore channel function, suggesting a potential therapeutic option for individuals with congenital stationary night blindness. Radhakrishnan et al. sought to determine the binding domain in the receptor retinol binding protein 4 receptor 2 (RBPR2) protein for the ligand retinol binding protein 4 (RBP4). RBP4 binds to all-*trans-*retinol (ROL) in the bloodstream and transports ROL to cells throughout the body. ROL is the major form of vitamin A within the bloodstream and serves as the precursor to 11-*cis* retinaldehyde, which is the vitamin A derivative essential for light detection. RBPR2 is a receptor for RBP4 and the absence of RBPR2 leads to vision loss, underscoring the importance of RBPR2 in maintaining ROL homeostasis within the eye. To better understand how RBPR2 binds to RBP4, the authors utilized molecular modeling and biochemical binding assays to interrogate specific amino acid changes in RPBR2 and to identify a critical binding domain for RBP4. Finally, Linnert et al. sought to identify interacting partners for ADGRV1 and CIB2, two proteins which when mutated cause Usher Syndrome. Using affinity proteomics and biochemical assays, the authors discovered that these USH proteins shared numerous interacting proteins and, unexpectedly, interacted with proteins of the Bardet Biedl Syndrome (BBS) complex. These data suggest that USH and BBS may share similar pathogenic mechanisms that lead to vision loss.

Given the clinical and genetic heterogeneity of IRDs, the development of regenerative medicine strategies could provide treatments that are not specific to a particular mutation or even a specific gene. To this end, Boyd et al. explore the role of cardiotrophin-like cytokine factor 1 (Clcf1) and cytokine receptor-like factor 1a (Crlf1a) to induce Muller glia to proliferate in the zebrafish retina. The zebrafish has an innate ability to regenerate retinal cells following injury. By leveraging knowledge of how this process is regulated in zebrafish, regenerative strategies for humans with IRDs may be developed. Other gene-agnostic approaches may include mitigating the effect of inflammation. Sarici et al. use retrospective clinical data to demonstrate that managing inflammation in IRDs can potentially mitigate disease progression regardless of the genetic mutation. Such efforts may prolong the window of opportunity for bespoke gene therapy interventions.

In conclusion, this Research Topic provides current insights into the complexities faced by clinicians and researchers in accurately diagnosing and investigating the pathomechanisms of IRDs. This Research Topic should benefit investigators interested in the latest advances in clinical genetics and the approaches used to investigate the functional consequences of genetic variants associated with IRDs.

